# Expression of target and reference genes in *Daphnia magna *exposed to ibuprofen

**DOI:** 10.1186/1471-2164-7-175

**Published:** 2006-07-07

**Authors:** Lars-Henrik Heckmann, Richard Connon, Thomas H Hutchinson, Steve J Maund, Richard M Sibly, Amanda Callaghan

**Affiliations:** 1 The University of Reading, School of Biological Sciences, Environmental Biology, PO Box 228, RG6 6AJ, UK; 2 AstraZeneca Global SHE, Brixham Environmental Laboratory, Devon, TQ5 8BA, UK; 3 Syngenta Crop Protection AG, 4002 Basel, Switzerland

## Abstract

**Background:**

Transcriptomic techniques are now being applied in ecotoxicology and toxicology to measure the impact of stressors and develop understanding of mechanisms of toxicity. Microarray technology in particular offers the potential to measure thousands of gene responses simultaneously. However, it is important that microarrays responses should be validated, at least initially, using real-time quantitative polymerase chain reaction (QPCR). The accurate measurement of target gene expression requires normalisation to an invariant internal control e.g., total RNA or reference genes. Reference genes are preferable, as they control for variation inherent in the cDNA synthesis and PCR. However, reference gene expression can vary between tissues and experimental conditions, which makes it crucial to validate them prior to application.

**Results:**

We evaluated 10 candidate reference genes for QPCR in *Daphnia magna *following a 24 h exposure to the non-steroidal anti-inflammatory drug (NSAID) ibuprofen (IB) at 0, 20, 40 and 80 mg IB l^-1^. Six of the 10 candidates appeared suitable for use as reference genes. As a robust approach, we used a combination normalisation factor (*NF*), calculated using the geNorm application, based on the geometric mean of three selected reference genes: *glyceraldehyde-3-phosphate dehydrogenase*, *ubiquitin conjugating enzyme *and *actin*. The effects of normalisation are illustrated using as target gene *leukotriene B4 12-hydroxydehydrogenase *(*Ltb4dh*), which was up-regulated following 24 h exposure to 63–81 mg IB l^-1^.

**Conclusions:**

As anticipated, use of the *NF *clarified the response of *Ltb4dh *in daphnids exposed to sublethal levels of ibuprofen. Our findings emphasise the importance in toxicogenomics of finding and applying invariant internal QPCR control(s) relevant to the study conditions.

## Background

Toxicogenomics is an evolving discipline investigating stressor impact at the genome level [1]. Microarrays are state-of-the-art tools for global gene expression profiling of the response of an organism in a particular biological context. However, expression levels of key genes responding on the microarray need to be validated [2]. The preferred technique of validation is by real-time quantitative polymerase chain reaction (QPCR) [3,4]. Although QPCR is a fairly reliable technique, amplification can vary depending on factors such as RNA integrity, reverse transcriptase (RT) efficiencies, sample-to-sample variations in amplification efficiency, and variation in cDNA sample loading. Using the same sample sizes, assessing RNA integrity and equalising RNA concentrations prior to RT are some of the basic normalisation steps in QPCR [5]. However, normalisation to some internal control is essential for accurate QPCR to balance sample-to-sample variations within the RT and PCR reactions. Internal control is generally achieved using reference genes, also known as housekeeping genes.

Some of the most commonly used reference genes, such as *β-actin *and *glyceraldehyde-3-phosphate dehydrogenase *(*GAPDH*), are historical carryovers from northern blotting, a "predecessor" of QPCR [5]. It is good practice to rigorously validate the suitability of these reference genes under the specific experimental condition to which they are applied [5-7]. Total RNA and rRNA have also been suggested as internal controls, although again differences between tissue, individuals and experimental conditions apply [8]. Moreover, using RNA as an internal control has the disadvantage of not controlling for variation inherent in the RT and PCR reactions [5]. None of these genes or RNA products are completely invariable, and thus each needs validation before use [5-7]. A further problem of using only one reference gene for normalisation, is that it theoretically should have been normalised itself prior to target gene normalisation [9]. Various methods have been proposed to overcome these problems. One involves the use of a normalisation factor (*NF*) based on the geometric mean of multiple carefully selected reference genes. This *NF *can be calculated by geNorm (see Vandesompele et al., 2002 [10] and the geNorm website [11]) or other freely available Excel (Microsoft) based software applications, e.g. BestKeeper [12] (see Pfaffl et al., 2004 [9]). Vandesompele et al., 2002 [10] recommend that *NF*s should be estimated from a minimum of three reference genes. The advantage of this approach is that it allows for the cumulative error of the entire process from RNA extraction to the QPCR.

Here we evaluate 10 candidate reference genes for QPCR in *Daphnia magna *following a 24 h exposure to the non-steroidal anti-inflammatory drug (NSAID) ibuprofen (IB); 20, 40 and 80 mg IB l^-1^. This concentration range represents a sublethal exposure based on our earlier work showing that the effect concentration needed to immobilise 50% (EC_50_) of the exposed individuals was 107.7 mg IB l^-1 ^following 48 h [13]. The tested candidate reference genes were chosen to cover a range of gene ontologies (Table 1, Methods), but mostly representing what may be considered as classical reference genes such as *actin *and *GAPDH *[6].

We use the geNorm algorithm [10] to estimate the variability of the reference genes, and to discover an optimal normalisation factor (*NF*), based on the geometric mean of three of them. geNorm estimates reference gene variability by calculating an expression level ratio for any two candidate reference genes. The variation between the expression ratios is then used as an inverse measure to estimate the variability of the analysed reference genes (please see Methods or Vandesompele et al., (2002) [10] for further information). The approach is illustrated using as target gene the *D. magna *ortholog of *leukotriene B4 12-hydroxydehydrogenase *(*Ltb4dh*), which was identified as being up-regulated in a suppressive subtractive hybridisation following 24 h exposure to 63–81 mg IB l^-1 ^[13]. *Ltb4dh *is of special interest because NSAIDs are known to inhibit the mammalian biosynthesis of various eicosanoids (e.g. prostaglandins, epoxyeicosatrienoic acids and leukotriene) that play important regulatory and signalling functions, for instance regulation of ion flux [14,15]. As anticipated, use of the *NF *clarified the response of *Ltb4dh *to ibuprofen.

## Results

### Water chemistry and quantification of ibuprofen

The chemical parameters pH and conductivity remained stable throughout the exposure, ranging between 7.8–7.9 and 422–452 μS cm^-1^, respectively. Water temperatures ranged from 20.8 to 21.0°C. Ibuprofen-sodium increased measured pH by approximately 9% and conductivity by approximately 7% in the highest treatment (80 mg IB l^-1^) compared with the control (data not shown).

Quantification of IB revealed that nominal concentrations were within ± 10% of the measured concentration; except for 20 mg IB l^-1 ^replicate three that was 10.6% lower than the nominal (data not shown). No mortality was observed in *D. magna *in any of the treatments following exposure.

### Relative expression and variability of candidate reference genes

Relative gene expression was estimated using DART-PCR (see Methods). There was a significant (*p *< 0.05) down-regulation of *Atb *and *Cyp *at 80 mg IB l^-1 ^compared with the control (Table 2). The eight candidate reference genes unaffected by the IB treatment were analysed by geNorm (see Methods) ranking the least variable genes as; *UBC *= *GAPDH *<*Act *<*WARS *<*SDH *<*TBP *<*18S *<*28S *(Table 2). Subsequently, geNorm was applied to estimate seven normalisation factors,*NF*_2 _to *NF*_8_, based on the geometric mean of the relative expression of the included reference genes. The geNorm algorithm allocates the two least variable genes, here *GAPDH *and *UBC*, to *NF*_2_. Then the sequential *NF*_3 _was based on the genes from *NF*_2 _and the third least variable gene *Act*, and so on.

### Variability of normalisation factors

**Figure 1:**
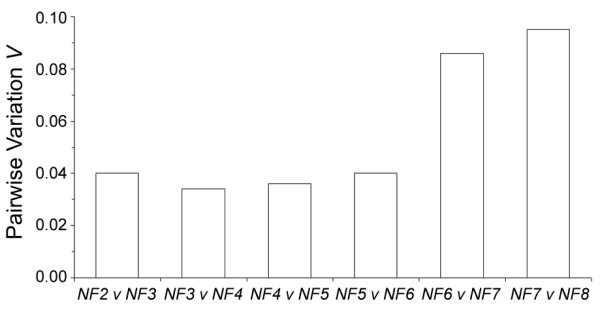
**Pairwise variation of sequential normalisation factors (*NF*_*n *_vs. *NF*_*n*+1_) estimated by geNorm**. *NF*_2 _is based on the geometric mean of *GAPDH *and *UBC*; *NF*_3 _is *NF*_2 _and *Act*; *NF*_4 _is *NF*_3 _and *WARS*; *NF*_5 _is *NF*_4 _and *SDH*; *NF*_6 _is *NF*_5 _and *TBP*; *NF*_7 _is *NF*_6 _and *28S*; *NF*_8 _is *NF*_7 _and *18S *(gene name abbreviations as Table 1).

Pairwise comparisons of sequential normalisation factors computed by geNorm revealed a similar level of variability between *NF*_2 _vs. *NF*_3_, *NF*_3 _vs. *NF*_4_, *NF*_4 _vs. *NF*_5 _and *NF*_5 _vs. *NF*_6 _(Fig. 1). This suggests that the use of either of these *NF*s as internal control would be valid. But pairwise comparisons of *NF*_6 _vs. *NF*_7 _and *NF*_7 _vs. *NF*_8_, (*28S *(*NF*_7_) and *18S *(*NF*_8_)), more than doubled the variability (Fig. 1). Vandesompele et al., 2002 [10] recommend using as few reference genes as feasible, but a minimum of three. Thus, although it gave a similar variability to other combinations, we chose *NF*_3_, (*GAPDH*, *UBC *and *Act*) since it gave an optimal *NF *with a low level of variation using the lowest possible number of reference genes.

### Relative expression of reference genes following normalisation

There is a circular problem with normalising a target gene with reference genes that should be normalised themselves prior to target gene normalisation [9]. Thus, in addition to normalising the target gene, we also normalised all 10 reference genes by *NF*_3_. *Atb *had the same significant (*p *< 0.05) differences as prior to normalisation, and should therefore be regarded as a target gene. None of the other reference genes, including *Cyp*, were significantly (*p *> 0.05) affected by IB following normalisation (data not shown).

### Relative expression of the target gene Ltb4dh following normalisation

**Figure 2:**
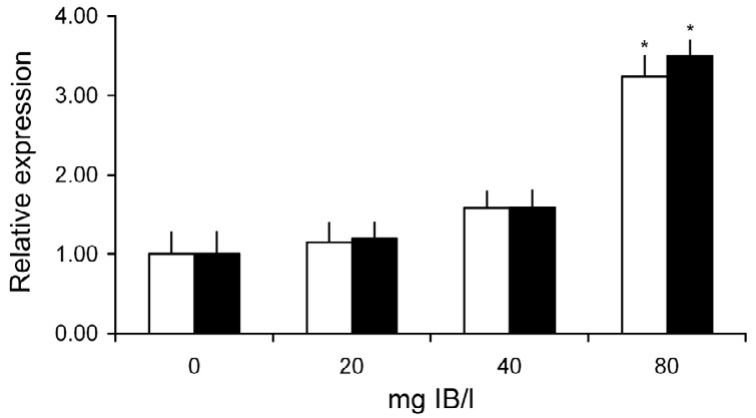
**Relative gene expression of target gene *Ltb4dh *in *Daphnia magna *following a 24 h exposure to ibuprofen**. Mean relative expression (*n *= 3) ± SD of target gene *Ltb4dh *(amplification efficiency E = 1.060 ± 0.042) without normalisation (white bars) or with normalisation (black bars) to normalisation factor *NF*_3 _based on the geometric mean of *Act*, *GAPDH *and *UBC *(gene name abbreviations as Table 1). Expression levels are displayed relative to the mean control level. An asterisk (*) indicates a significant difference (*p *< 0.05) between the highest treatment and any other treatment.

**Figure 3:**
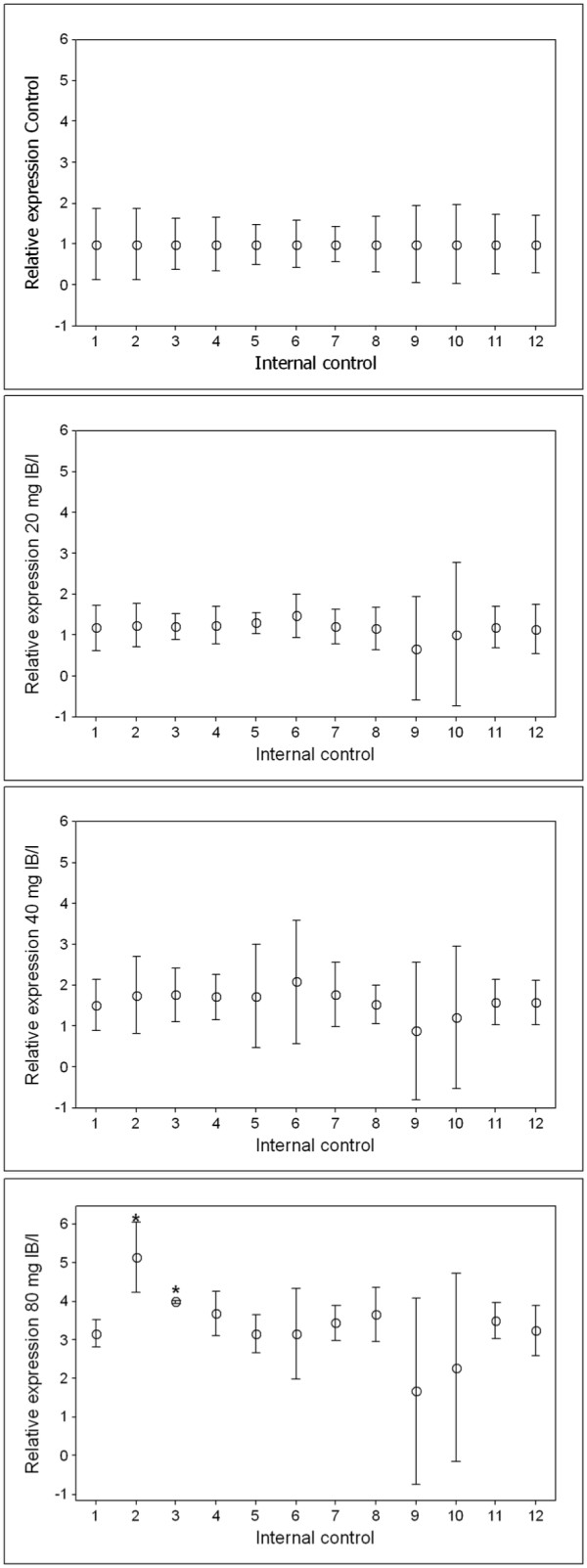
**Relative gene expression of target gene *Ltb4dh *in *Daphnia magna *following a 24 h exposure to ibuprofen**. Mean relative expression (*n *= 3) ± SD of *Ltb4dh *following normalisation to 10 candidate reference genes and a normalisation factor (*NF*). Expression levels are displayed relative to the mean control level. For comparison, the normalised relative expression of *Ltb4dh *is displayed by treatment group. Relative expression of *Ltb4dh *normalised to: *Act *(1); *Atb *(2); *Cyp *(3); *GAPDH *(4); *SDH *(5); *TBP *(6); *WARS *(7); *UBC *(8); *18S *(9); *28S *(10); *NF*_3 _based on the geometric mean of *Act*, *GAPDH *and *UBC *(11); and *Ltb4dh *without normalisation (12) (gene name abbreviations as Table 1). An asterisk (*) indicates a significant difference (*p *< 0.05) from *Ltb4dh *normalised by *NF*_3_.

Without normalisation, the target gene *Ltb4dh *was significantly (*p *< 0.05) up-regulated at 80 mg IB l^-1 ^compared with any of the other treatment groups having a 3.24-fold higher expression than the control (Fig. 2). The relative expression of *Ltb4dh *followed a significant (*p *< 0.05) dose-dependent relationship (data not shown). Normalisation with *NF*_3 _produced a similar expression (Fig. 2). The relative expression of *Ltb4dh *was normalised individually by each of the 10 candidate reference genes and by *NF*_3 _to assess how their variability influenced target gene expression. Overall, the mean fold difference in relative expression between 80 mg IB l^-1 ^and the control was 3.34 ± 0.85 SD ranging from 1.66 (*18S*) to 5.15 (*Atb*) (Fig. 3). Target gene expression normalised to any of the reference genes was compared to normalisation with *NF*_3 _revealing that there was a significant (*p *< 0.05) difference within the 80 mg IB l^-1 ^treatment between normalisation of *Ltb4dh *to *Atb *and *Cyp *respectively, and *Ltb4dh *normalised to *NF*_3 _(Fig. 3). Although not significant (*p *> 0.05), normalisation of *Ltb4dh *to either *18S *or *28S *caused a large increase in the variation of target gene expression in any of the IB treatments (Fig. 3). Both rRNA genes reduced significant differences observed in *Ltb4dh*, with or without normalisation to *NF*_3 _(data not shown).

## Discussion

We investigated the variability of 10 candidate reference genes in *D. magna *following a 24 h exposure to IB to discover the least variable internal control(s) for QPCR normalisation. A comparison of the reference genes, using the geNorm software, ranked the least variable genes as; *UBC *= *GAPDH *<*Act *<*WARS *<*SDH *<*TBP *<*18S *<*28S*. *Atb *and *Cyp *were not included in the geNorm analysis as they were down-regulated by IB. Furthermore, geNorm identified the optimal normalisation factor as *NF*_3 _based on the geometric mean expression of *UBC*, *GAPDH *and *Act*. This *NF *was based on the lowest recommended number of reference genes with the lowest level of variation [10]. The response to IB of an example target gene, *Ltb4dh*, was little changed by application of *NF*_3 _(Fig. 2), although at the highest concentration of IB, there was a slight increase in the response, and a diminution of the variation between replicates. Such reduction in the variation between replicates is exactly what one hopes to achieve through normalisation.

QPCR confirmed that *Ltb4dh *was up-regulated following exposure to IB [13]. NSAIDs are known to inhibit the biosynthesis of various eicosanoids that play important regulatory and signalling functions, e.g. regulation of ion flux. In mammals, *Ltb4dh *is involved in the metabolism of leukotriene B_4 _an eicosanoid that is formed in the lipoxygenase pathway [15]. There is evidence of eicosanoid pathways in invertebrates being similar to the mammalian pathways [16]. Further experimentation, involving a global expression profile based on cDNA microarrays, is underway to support this hypothesis and reveal the overall molecular stress response of *D. magna *to IB. To our knowledge, this is one of the first genomic studies to look at the impact of a NSAID on an aquatic invertebrate. The only non-mammalian studies are on yeast *Saccharomyces cerevisiae *[17], a *Drosophila *cell line [18] and *Bacillus megaterium *[19].

Following normalisation to *NF*_3 _it transpired that *Atb*, assayed as a candidate reference gene, was affected by IB, and so should be regarded as an IB target gene. In a review on the molecular mechanism of NSAIDs in mammals, Tarnawski and Jones, 2003 [20] reveal that it is likely that NSAIDs exert many inhibitory effects, one of which is the inhibition of cell division. The observed suppression of *Atb *following exposure to IB could be linked to this, since alpha-tubulin helps form microtubules that are involved in cell division, cell structure, and transportation of vesicles and organelles.

Of the remaining candidate reference genes, *Act*, *GAPDH*, *UBC*, *WARS*, *SDH *and *TBP *had a constant expression following exposure to IB. But although, *GAPDH *and *UBC *were the most stable reference genes they both had a weakly significant (*p *< 0.10) linear trend of down-regulation. Thus, relying solely on either of these two reference genes for normalisation could obscure the output.

Using reference genes as an internal control in QPCR is currently the recommended approach [5]. However, Bustin and Nolan, 2004 [21] advocate the introduction of more standard analysis and reporting procedures in QPCR similar to the MIAME guidelines [22] established for microarray technology. In union with other authors [5,10,21,23] we would like to recommend some basic applications for QPCR: (i) apply a dissociation curve in every QPCR, and omit any samples diverging from the curve; (ii) use a minimum of two non-template controls (NTCs) in every QPCR, and omit any samples having a cycle threshold (C_t_) value close to or higher than the mean NTC C_t_-value; (iii) avoid inter-assay comparison if possible, as it is more appropriate to duplicate the samples in one QPCR than using triplicates of each sample, but having to run two QPCRs (S.N. Peirson, personal communication); (iv) use a *NF *based on a minimum of three validated reference genes for target gene normalisation [10]; (v) integrating the DART-PCR approach [23], which verifies sample-to-sample variations in amplification efficiency with a normalisation factor software package, e.g. geNorm [10], creates a powerful tool for analysing relative gene expression of target genes.

## Conclusions

Investigators engaging in toxicogenomic research using invertebrates, or vertebrate cell lines, exposed to NSAIDs may be able to apply our findings directly under similar experimental conditions, e.g. acute exposure. However, we strongly recommend validating reference genes prior to commencing target gene expression profiling under experimental conditions different than those described herein. Our findings stress the importance of performing fundamental research, by validating the most invariant internal QPCR control(s) of a particular experimental condition, prior to investigating the expression of target genes examined by QPCR only, or as QPCR validation of microarray data.

## Methods

### Test species

*Daphnia magna *Straus (Clone Type 5 – IRCHA), originally obtained from the Water Research Centre (WRc), Medmenham, UK, were investigated. For further information on culturing conditions of *D. magna *please see Hooper et al., 2006 [24].

### Experimental design and analytical chemistry

The experimental design consisted of three replicates of a control and three treatments with ibuprofen-sodium (Sigma-Aldrich, CAS no. 31121-93-4) containing 20, 40 and 80 mg IB l^-1^, respectively. Each replicate consisted of 100 third brood neonates (< 24 h old) placed in a 1000 ml glass beaker containing one litre of reconstituted freshwater with or without the addition of IB. Neonates were exposed to IB for 24 h under the same conditions as stock cultures (see Hooper et al., 2006 [24]), but without feeding. To quantify IB, 1.5 ml was sampled from each replicate at 0 h and 24 h and stored at -20°C. Subsequently, the free base concentration of IB was measured at 217 nm by UV-spectrophotometry using an Ultrospec3000 (Biochrom) after the method of Pascoe et al., 2003 [25]. Conductivity, pH and water temperature were checked at the beginning and at the end of the exposure to verify stable water chemistry.

### Tissue preparation, RNA extraction and reverse transcription

Following exposure, the neonates were immediately placed in 0.2 ml of RNA*later*^® ^(Ambion) and stored at -80°C. Subsequently, total RNA was extracted using the RNeasy Mini kit with on-column DNase treatment (Qiagen) to remove any traces of genomic DNA following the manufacturer's instructions. RNA concentrations were determined by spectrophotometry using GeneQuant Pro (Biochrom). The integrity of the RNA was verified by 1% agarose gel electrophoresis. Four micrograms of DNase-treated total RNA was reverse transcribed with Oligo(dT)_12–18 _primers (Invitrogen) using the Omniscript Reverse Transcriptase kit (Qiagen) following the manufacturer's instructions. Once synthesised, cDNA was diluted 10-fold resulting in total RNA concentrations of 10 ng/μl and stored at -20°C.

### Real-time quantitative PCR

Ten expressed sequence tags (ESTs) were obtained from the NCBI website [26] and the *Daphnia Genomics Consortium *website [27] (see Colbourne et al., 2005 [28]). ESTs were verified by BLASTN and BLASTX analysis [26]. Primers were designed using Primer3 [29], and synthesised by MWG. The investigated ESTs, accession numbers, primer sequences and gene ontology are shown in Table 1.

QPCR was conducted on the GeneAmp 5700 Sequence Detection System (SDS) (Applied Biosystems) using ABsolute™ QPCR SYBR^® ^Green ROX (500 nM) mix (ABgene). Each reaction was run in triplicate and contained 2.5 μl of cDNA template (equivalent to 25 ng total RNA) along with 900 nM primers in a final volume of 25 μl. Cycling parameters were 95°C for 15 min to activate the DNA polymerase, then 40 cycles of 95°C for 15 s and 60°C for 1 min. Melting curves were performed by using dissociation curve SDS software version 1.3 (Applied Biosystems) to verify that only a single product with no primer-dimers were amplified.

### Data analysis and statistics

Following QPCR, the raw data was exported to an Excel workbook (Microsoft), entitled Data Analysis for Real-Time PCR (DART-PCR) [23]. This programmed workbook enables calculation of threshold cycles and amplification efficiencies for every sample. Differences in amplification efficiency were assessed using one-way analysis of variance (ANOVA), based upon the null hypotheses: (i) that amplification efficiency is comparable within sample groups (outlier detection) and (ii) that amplification efficiency is comparable between sample groups (amplification equivalence) [23]. Outliers identified by DART-PCR and samples diverging from the dissociation curve were omitted prior to further analysis.

Differences in relative expression were assessed on log-transformed data using ANOVA and Tukey's honestly significant difference (HSD) for post hoc comparisons. Equality of variance was tested using Levene's test. The relative expression of the reference genes calculated by DART-PCR was exported as an Excel workbook (Microsoft) to geNorm version 3.4 [10]. The "geNorm" algorithm is based on the proposition that, in any set of assays, the expression levels of suitable reference genes should be perfectly correlated. However, because of technical difficulties in assessing correlation in circumstances when there is little variation, a modified procedure is adopted as follows [10]. First, the ratio of expression levels is calculated for any two candidate reference genes. Assuming that the expression ratios should not vary between assays, the variation between the expression ratios is used as an inverse measure, here referred to as gene variability, of the adequacy of the reference genes to be used for normalisation. Thus, the lower the variation, the better the reference genes. Variation is here measured as standard deviation. Given a set of assays that covers the treatments of interest, this procedure can be used to identify the best two reference genes from all possible pairs of reference genes available to the experimenter. A normalisation factor (*NF*_2_) is then produced for these two genes, calculated as the geometric mean of their expression levels. The procedure is then extended to assess whether stepwise inclusion of additional reference genes – producing *NF*_3 _to *NF*_*n *_– will reduce the average pairwise variation between them. The optimal number of reference genes to be included in a *NF *is estimated by comparing the pairwise variation between sequential normalisation factors [10], e.g. *NF*_3 _vs. *NF*_4_. All statistical tests were performed in SPSS 12.0.1 for Windows (SPSS, Chicago, IL, USA) applying a significant level of 5%, except reference gene variability and *NF *analysis, which was computed in geNorm version 3.4 [10].

## Authors' contributions

All authors participated in the coordination and design of the study. LHH performed all the practical aspects of the study (i.e. exposure of *D. magna *and genomic analyses) under the supervision of AC and RC. Furthermore, LHH performed the statistical analyses under supervision of RMS, and drafted the manuscript. All authors read, contributed intellectually and approved the final manuscript.

## Figures and Tables

**Table 1:** Candidate reference genes and target gene investigated

**Gene name**	**Symbol**	**GenBank accession no.**	**wFleaBase EST no.**	**Gene Ontology**^a^	**Forward primer (5'-3')**	**Reverse primer (5'-3')**	**Amplicon size (bp)**
*Actin*	*Act*	AJ292554		Structural constituent of cytoskeleton (F) GO:0005200	CCA CAC TGT CCC CAT TTA TGA A	CGC GAC CAG CCA AAT CC	71
*Alpha-tubulin*	*Atb*		WFes0007807	Microtubule (C) GO:0005874	TGG AGG TGG TGA CGA CT	CCA AGT CGA CAA AGA CAG CA	89
*Cyclophilin*	*Cyp*		WFes0012034	Protein folding (P) GO:0006457	GAC TTT CCA CCA GTG CCA TT	AAC TTT CCA TCG CAT CAT CC	78
*Glyceraldehyde-3- phosphate dehydrogenase*	*GAPDH*	AJ292555		Glycolysis (P) GO:0006096	GGC AAG CTA GTT GTC AAT GG	TAT TCA GCT CCA GCA GTT CC	89
*Succinate dehydrogenase*	*SDH*		WFes0009191	Tricarboxylic acid cycle (P) GO:0006099	TGC CAT TTA GTC GCA CTC AG	GTG AGC TTG TCC TCC TTT GC	89
*TATA-box binding protein*	*TBP*		WFes0002485	Transcription initiation (P) GO:0006367	GCA GGG AAG TTT AGT TTC TGG A	TGG TAT GCA CAG GAG CAA AG	88
*Tryptophanyl- tRNA synthetase*	*WARS*		WFes0003968	Tryptophanyl-tRNA ligase activity (F) GO:0004830	GCA CTG TGA TCA GCT CTT GC	TTT CGA TTG GAG GAA ATT CG	82
*Ubiquitin Conjugating Enzyme*	*UBC*		WFes0004602	Ubiquitin-dependent protein catabolism (P) GO:0006511	TCA CCT GCA CTC ACC ATT TC	AAT CTC CGG AAC CAA AGG AT	90
*18S ribosomal RNA*	*18S*	AF070104		Protein biosynthesis (P) GO:0006412	CGC TCT GAA TCA AGG GTG TT	TGT CCG ACC GTG AAG AGA GT	77
*28S ribosomal RNA*	*28S*	AF532883		Protein biosynthesis (P) GO:0006412	GAG GCG CAA TGA AAG TGA AG	TGT TCG AGA CGG GAT CA	70
*Leukotriene B4 12-hydroxy- dehydrogenase*^b^	*Ltb4dh*	DY242190		Response to toxin (P) GO:0009636	AAC CTA CAC CGA GGG TTT CG	TCC AAC ATT AAC GCC ATT AAG C	70

^a^Several of the candidate reference genes are classified within multiple biological processes (P), molecular functions (F) or associated with one or more cellular components (C). A biological function, accompanied by a GO number, has been listed here for each gene (for more information visit ).

^b^Target gene also known as 15-oxoprostaglandin 13-reductase or more generally as NADP(H)-dependent alkenal/one oxidoreductase

**Table 2:** Relative gene expression and variability of candidate reference genes

	**Relative expression**^a^					
**Gene**	**Control**	**20 mg IB l**^-1^	**40 mg IB l**^-1^	**80 mg IB l**^-1^	**Gene variability**^b^	**Amplification efficiency (E)**^a^
*Act*	1.000 ± 0.078	0.963 ± 0.035	1.030 ± 0.044	1.001 ± 0.040	0.119	0.795 ± 0.066
*Atb*	1.000 ± 0.129	0.896 ± 0.126	0.885 ± 0.095	**0.613 ± 0.045**^c^	-	1.010 ± 0.016
*Cyp*	1.000 ± 0.040	0.949 ± 0.103	0.900 ± 0.015	**0.817 ± 0.067**^d^	-	1.038 ± 0.016
*GAPDH*	1.000 ± 0.093	0.914 ± 0.059	0.919 ± 0.047	0.879 ± 0.067	0.098	0.946 ± 0.029
*SDH*	1.000 ± 0.145	0.889 ± 0.170	0.950 ± 0.180	1.027 ± 0.026	0.160	0.992 ± 0.015
*TBP*	1.000 ± 0.096	0.786 ± 0.176	0.788 ± 0.153	1.038 ± 0.084	0.192	0.925 ± 0.016
*WARS*	1.000 ± 0.117	0.954 ± 0.098	0.906 ± 0.060	0.955 ± 0.081	0.136	0.922 ± 0.064
*UBC*	1.000 ± 0.040	0.995 ± 0.095	1.024 ± 0.016	0.888 ± 0.097	0.098	1.034 ± 0.013
*18S*	1.000 ± 0.279	2.096 ± 1.074	2.506 ± 1.640	2.204 ± 0.910	0.430	0.853 ± 0.089
*28S*	1.000 ± 0.261	1.247 ± 0.445	1.410 ± 0.560	1.457 ± 0.616	0.313	0.846 ± 0.020

Gene name abbreviations as Table 1.

^a^Values are mean ± SD (IB refers to ibuprofen). Expression levels are displayed relative to the mean control level.

^b^Gene variability is estimated by geNorm as the variation (SD) of a given reference gene relative to the remaining reference genes (*Atb *and *Cyp *are not included in the analysis as they are significantly down-regulated by IB).

^c^Highest treatment (80 mg IB l^-1^) significantly different from any other treatment (*p *< 0.05).

^d^Highest treatment (80 mg IB l^-1^) significantly different from control (*p *< 0.05).
